# Use of a novel supplementary food and measures to control inflammation in malnourished pregnant women in Sierra Leone to improve birth outcomes: study protocol for a prospective, randomized, controlled clinical effectiveness trial

**DOI:** 10.1186/s40795-018-0218-y

**Published:** 2018-04-02

**Authors:** D. Taylor Hendrixson, Aminata Shamit Koroma, Meghan Callaghan-Gillespie, Jacklyn Weber, Peggy Papathakis, Mark J. Manary

**Affiliations:** 1 Department of Pediatrics, One Children’s Place, Washington University in St. Louis, Campus Box 8116, Saint Louis, MO 63110 USA; 2 Ministry of Health and Sanitation, The Republic of Sierra Leone, 4th Floor Youyi Building, Freetown, Sierra Leone; 3 Department of Food Science and Nutrition, California Polytechnic State University, San Luis Obispo, CA 93407 USA

**Keywords:** Supplementary foods, Intrauterine growth restriction, Legumes, Low birth weight, Malnutrition, Pregnancy, Stunting, RUSF

## Abstract

**Background:**

The negative synergy between poor nutritional status and infectious diseases is doubly detrimental in pregnancy. In Sierra Leone, maternal malnutrition is amongst the highest in the world, while maternal mortality is high at 1320/100,000 live births and stunting in under-five is 37.9%, ranked 110/132 worldwide. Maternal malnutrition has been associated with preterm birth, small-for-gestational age infants, and poor maternal outcomes. Infants born prematurely or small-for-gestational age experience higher mortality and are at risk for stunting and decreased cognitive performance. Nutritional interventions alone during pregnancy may not be as effective in the setting of increased inflammation from repeated infections. Interventions are needed to improve maternal outcomes and reduce stunting in this population.

**Methods/design:**

This will be a prospective, randomized, controlled clinical effectiveness trial of an improved supplementary food plus anti-infective therapies compared to standard therapy in malnourished pregnant women. Pregnant women will be randomized to receive a low water activity, ready-to-use supplementary food plus five anti-infective interventions or the standard of care which is 3.5 kg corn/ soy blended flour with 350 mL vegetable oil every two weeks. The five anti-infective interventions are 1) insecticide-treated mosquito net at the time of enrollment into the study, 2) sulfadoxine-pyrimethamine given every 4 weeks, beginning at enrollment or at 13 weeks’ gestation, whichever is later, 3)azithromycin at a dose of 1 g given once at enrollment (after first trimester)and again during 28–34 weeks of gestation, 4)single dose 400 mg albendazole given in second trimester, and 5) testing and treatment for bacterial vaginosis at enrollment and again at 28–34 weeks of gestation. Treatment will be provided for the duration of the pregnancy. The primary outcome measure will be birth length. Secondary outcomes in the mothers will include rates of maternal weight gain and increase in mid-upper arm circumference, and time to maternal anthropometric recovery. Secondary outcomes in the infants will include birth weight, birth head circumference, and linear and ponderal growth.

**Discussion:**

Malnutrition remains a major problem in the developing world with lasting maternal and infant consequences. Maternal malnutrition has been associated with intrauterine growth retardation, low birth weight (LBW), pre-term delivery and poor cognitive development. Nutritional interventions alone have not been successful in reducing stunting. By bundling nutritional and anti-infective interventions, we aim to reduce intrauterine growth restriction and low birth weight in moderately malnourished pregnant women in Sierra Leone. If successful, this bundle can easily be implemented by governments or non-governmental organizations.

**Trial registration:**

Clinicaltrials.gov NCT03079388; Date: March 5, 2017.

## Background

### Maternal malnutrition

Acute malnutrition in pregnancy is a risk factor for adverse outcomes in mothers and their unborn children. There is no universally endorsed method of identification of maternal malnutrition, though a low mid-upper arm circumference (MUAC) has been used reproducibly in a standardized manner [1]. Maternal undernutrition/malnutrition impairs placental formation and results in reduction of placental size, alterations in histomorphology and reduction of blood flow, which can diminish nutrient delivery to the fetus [2]. Maternal malnutrition during pregnancy has been linked to intrauterine growth retardation, low birth weight (LBW), pre-term delivery and poor cognitive development in offspring. Both preterm infants and small-for-gestational-age infants have increased risk of mortality during the neonatal period and extending into early childhood [3]. The long term consequences include limited academic performance, stunted professional achievement, and lower wages as adults [4, 5]. Additionally, undernutrition during pregnancy may result life-threatening hemorrhage, increased risk for sepsis, hypertensive disorders of pregnancy and increased all-cause mortality [6–8]. In Sierra Leone, maternal anemia is amongst the highest in the world with over 50% of pregnant women affected [9]. Maternal mortality is extremely high at 1360/100,000 live births, and stunting in under-five children is 37.9%, ranked 110/132 worldwide [10].

Given the multiple adverse effects of maternal undernutrition, interventions early in pregnancy to improve nutritional status and prevent these outcomes are needed. There have been several trials conducted with aims of improving maternal malnutrition and improving birth outcomes. A large meta-analysis in 2012 including 12 trials demonstrated that pregnant women who received balanced energy and protein supplementation had a significant reduction in stillbirth and risk of small-for-gestational-age infants, as well as, a significant increase in birth weight [11]. Additionally, a second large meta-analysis in 2015 including 15 trials antenatal interventions demonstrated that maternal micronutrient supplementation with iron and folic acid resulted in a significant decrease in the rate of stillbirth, low birthweight infants, and small-for-gestational age infants [12]. Antenatal maternal micronutrient supplementation may, also, result in improved body weight, MUAC, and head circumference persisting into early childhood [13]. Notably, these interventions have not demonstrated any significant effect on birth length or stunting.

In Sierra Leone districts with stunting rates > 40%, the national policy is to provide malnourished pregnant women a daily ration of 250 g corn-soy blend with sugar, 20 ml of oil, 60 mg iron and 400 μg folic acid supplement, from diagnosis until 6 months post-partum; however, coverage and scale-up of this program is limited and has only reached select districts.

### Maternal anemia

Anemia is highly prevalent in women in Sierra Leone at 45% in women of child bearing age. The highest prevalence of 50% is found among women age 15–19 [9]. Anemia during pregnancy may be attributed to poor dietary intake, inadequate absorption and blood loss from intestinal helminths or malaria [14]. The WHO recommends daily oral iron supplementation of 30-60 mg of elemental iron as part of routine antenatal care to reduce the risk of maternal anemia and low birth weight infants [15].

In addition to iron supplementation, preventative treatment of helminth infections has been shown as successful intervention during pregnancy [16–18]. Deworming with albendazole is safe, treats common helminth infections and has been shown to reduce anemia in pregnancy, especially when added to iron supplementation [18]. Additionally, preventing helminth infections is important as infections during pregnancy have been associated with lower cognitive and gross motor functioning in offspring [19]. Deworming of pregnant women should be delivered during antenatal visits; however, only 36% of pregnant women in Sierra Leone are dewormed [9].

### Inflammation during pregnancy

A recent trial in rural Malawi of a lipid-based nutritional supplement for pregnant women and its effect on newborn size did not demonstrate statistically significant difference between groups in the incidence of low birthweight, small-for-gestational age, preterm birth, or newborn stunting [20]. This may be because nutritional interventions for malnutrition may be less effective under conditions with excessive inflammation and infection, and especially so during pregnancy. Undernutrition impairs immune function, prolonging the inflammatory process, which subsequently depletes nutrient supply; therefore, without specifically addressing treatment for infections, undernourished mothers may be less responsive to nutritional interventions. The increased nutrient demands during pregnancy result in a heightened risk for malnutrition and infections for both mother and fetus. Women in Sierra Leone are subject to continuous malaria transmission, as well as poor sanitation and hygiene, with only 5% having access to piped water and 38% having access to improved sanitation [9].

Common infections in Sierra Leone and sub-Saharan Africa include malaria, sexually transmitted diseases and gastrointestinal parasite infestation. Pregnancy is a time when the immune system is less able to recognize and respond to infections, in part because greater immuno-tolerance is needed for a successful pregnancy. The placenta is an organ within which the immune response is blunted [21, 22].

Malaria is the most common cause of illness and death in Sierra Leone [9]. Malaria due to *Plasmodium falciparum*, in particular, is known to be especially damaging in pregnancy due to its high tropism for the placenta, leading to high rates of stillbirth, IUGR, and premature delivery [23, 24]. Ensuring that all pregnant women receive insecticide-treated nets (ITN) as early in pregnancy as possible is also an important component of keeping pregnant women safe from malaria [25]. The World Health Organization recommends the Intermittent Preventive Treatment of malaria during pregnancy (IPTp) which requires that all women residing in malaria endemic regions receive monthly doses of malaria chemoprophylaxis with sulfadoxine-pyrimethamine (SP) during the second and third trimesters of pregnancy [26]. This recommendation for monthly chemoprophylaxis supersedes the prior recommendation of two doses during pregnancy, as it has been shown to be safe and superior in reducing the risk of adverse pregnancy outcomes, even in areas where in vitro SP resistance is widespread [27, 28]. In Sierra Leone IPTp and the distribution of ITNs coverage for pregnant women needs strengthening with only about 41% of women following recommendation [9].

The most recent demographic health survey in Sierra Leone revealed that among women of reproductive age, 22.7% reported having an STI, genital discharge, and/or a genital sore/ulcer, and 26% did not get any treatment [9]. Azithromycin is a safe, broad spectrum antibiotic with activity against several common sexually transmitted diseases including chlamydia, gonorrhea, and chancroid, in addition to having some antimalarial activity. Given twice during pregnancy, azithromycin has been shown to reduce stunting and premature delivery in Malawi [29].

Among the sexually transmitted diseases, bacterial vaginosis has been associated with preterm labor and other adverse pregnancy outcomes. One large study has demonstrated that screening and treatment for bacterial vaginosis significantly reduces rates of late miscarriages [30]. Treatment of symptomatic pregnant woman is widely recommended. Routine screening and treatment of asymptomatic pregnant women remains controversial as a 2013 Cochrane review demonstrated no significant reduction in the odds of preterm birth in women receiving treatment [31]. This may not apply to malnourished women who have ongoing inflammation and an increased risk for preterm birth as there is evidence to support routine testing and treatment of both symptomatic and asymptomatic women in populations at high risk for preterm delivery [32, 33].

## Methods/design

### Study goals

In this study, we aim to determine whether a combined intervention of a specialized nutritious food for pregnant women and a package of anti-inflammatory interventions results in improved birth anthropometry compared with the standard of care.

### Study design

**Fig. 1:**
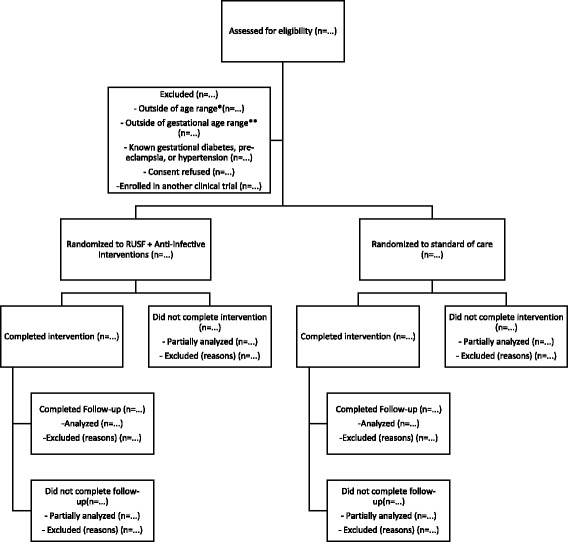
Patient flow diagram for clinical trial. *Women 16 years of age or older will be enrolled and followed until 6 months post-partum. **Women less than 35 weeks of gestation will be enrolled

This will be a prospective, randomized, controlled clinical effectiveness trial in pregnant women with malnutrition comparing the impact of combined nutrition and infection control interventions with the standard of care in Sierra Leone. The study will enroll pregnant women at less than 35 weeks of gestation with moderate or severe malnutrition. A probable patient diagram is shown in Fig. 1.

### Study location

The study will enroll patients at twenty antenatal clinics in rural Pujehun District in southern Sierra Leone and 10 antenatal clinics in the Rural Western Area District. In 2013, Pujehun District had the highest rate of under 5 stunting at 46.4% which is 8.4% above the national rate. Mothers who are thin (BMI < 18 kg/m^2^) are more likely to have children that are stunted, identifying Pujehun as an ideal district for a maternal nutrition intervention. The population predominantly belongs to the Mende ethnic group. Plantation farming is a major economic activity in the region with agricultural production of palm oil, cassava, and sweet potatoes [34]. Pujehun is one of the country’s poorest and least developed districts.

### Ethical considerations

We aim to complete the study using the highest ethical standards for clinical trials research in accordance with the Declaration of Helsinki [35]. Ethical approval has been obtained from the Sierra Leone Ethics and Scientific Review Committee (SLESRC) and from the Human Research Protection Office at Washington University in St. Louis. Support has also been obtained from Pujehun District Health Officer. Each subject will be briefed on the study procedures and interventions. Women older than 16 years of age will be eligible to consent for themselves. If a woman younger than the age of 16 desires to participate, consent will be obtained from the parent or guardian. Both verbal and written informed consent will be obtained from all enrollees. Those who are unable to sign or write their names will be asked to document consent using thumbprints.

### Eligibility criteria

Pregnant women presenting to any of the thirty selected antenatal clinics will be screened for enrolment eligibility. Women who have a fundal height no greater than 35 cm, MUAC ≤23 cm, and planning to reside in the study area and attend the antenatal clinic will be considered eligible. MUAC is a reproducible, reliable measure in the field and easily understandable by both health workers and participants. Fundal height will be used as a measure of gestational age. Exclusion criteria will include pregnant women less than 16 years of age who do not have an adult community member willing to consent for them or known complications of pregnancy such as gestational diabetes, pre-eclampsia, hypertension, or severe anemia.

### Outcome measures

The primary outcome measure of interest will be birth length in centimeters. The secondary outcome measures of interests include the total and average weekly maternal weight gain, recovery from maternal malnutrition identified as a MUAC > 23, newborn head circumference, birth weight, infant linear and ponderal growth, and infant survival at 3 months and 6 months.

### Sample size

The sample size will be 1514 pregnant women with malnutrition, divided equally between RUSF and control groups. This sample size allows for a 20% drop out rate and/or exclusion rate due to fundal height, leaving a final sample size of 1200 (600 per group) with a two tailed significance of 0.05, power of 80%. This will allow for detection of a difference of 0.22 SD or 0.5 cm in birth length and a difference of 0.19 SD or 80 g in birth weight.

### Randomization and blinding

Subjects will be randomized to receive the RUSF or the standard of care (supercereal, oil, and iron and folic acid tablets), using a random number generator which prospectively assigns participants to a study group. Since RUSF is visually distinct from flour, neither the study subjects nor the research study team members working directly with participants will be blinded. Study managers will be blinded to treatment during data analysis.

### Subject participation and data collection

A study team member will attend each participating antenatal clinic for subject recruitment. All women attending the antenatal clinic will be briefly informed of the study and entry criteria in a group setting. Women willing to be measured and potentially participate will be offered measurement of MUAC and screened for study eligibility.

All women meeting eligibility criteria will be offered enrollment and agree via informed consent. Informed consent will be obtained by the on-site study coordinator. All participants who are 16 years of age or older will be able to consent for themselves as adults. If a participant who is younger than 16 wants to participate in the study, they will be required to have consent from a parent or guardian. Upon enrollment, the participant will be interviewed. Demographic information will be recorded, as well as, time of last menses, estimated date of delivery, and fundal height. Anthropometry will include current weight, height, MUAC, and weight history if available. Available medical records of the women will be reviewed with attention to any current or previous pregnancy complications and medications. Subjects will answer a standard clinical symptoms questionnaire, a food frequency questionnaire, and a Household Food Insecurity Access Scale (HFIAS).

Participants will return for follow-up every 2 weeks for measurements and provision of food until delivery. Since birth measurements are primary outcomes, a birth weight team will be identified and trained in order to obtain accurate birth measurements within 48 h of delivery.

Participation in the study will continue throughout pregnancy, and follow-up with the mother and infant will continue until 6 months postpartum. Mothers and infants will be followed at usual infant clinic follow-up at 6 weeks, 3 months, and 6 months post-partum contact point clinic. Infant weight, linear growth, and morbidity will be monitored. Maternal weight, MUAC and morbidity will be assessed at these visits, as well.

### Data monitoring

The primary investigator will be responsible for the overall management of the trial. The on-site investigator will be responsible for day to day management of clinic sites. Adverse events have been defined as any reactions to RUSF, emesis, diarrhea, or rashes suggestive of food allergy.

To monitor the safety of participants, a data safety monitoring meeting will be held every other month. The meeting will be convened by the Director of Nutrition for the Ministry of Health and Sanitation for the government of Sierra Leone. Present at this meeting will be the director, a study nurse, the district health officer, and one of the investigators. At the meeting routine morbidity data, enrollment data, and participant satisfaction will be reviewed. Minutes of these meetings will be recorded and forwarded to the Sierra Leone Ethics and Scientific Review Committee (SLESRC).

A special inquiry will be convened whenever a serious adverse event is discovered. At any time a study staff member is informed about an unexpected event of a participant by a health surveillance assistant, national clinic staff, or community member, this will be reported to the Director of Nutrition for the Ministry of Health and Sanitation for the government of Sierra Leone within 24 h. Information will be gathered, and a summary of the event will be given to the data safety monitoring board within 24 h of being informed. The event will be reviewed in detail by the principle investigator and if the information supports the concern that this could be study related, an independent physician will be asked to review the case and render an opinion. The findings and conclusions will be recorded and forward to SLESRC and the Washington University IRB.

### Interventions for malnutrition during pregnancy

#### Ready-to-use supplementary food

Linear programming (LP) technology, a formulaic computer database program listing all potential ingredients, nutritional composition, prices, and country specific availability for Sierra Leone has been used to develop and optimize the RUSF. The RUSF will be produced by Project Peanut Butter (PPB), Sierra Leone, a local non-profit organization that produces and distributes ready to use therapeutic foods. PPB operates a factory in Freetown, Sierra Leone that has been internationally certified to supply RUSF to UN agencies.

The spreadsheet-based tool identified a low-cost formula for RUSF for pregnant women. The tool allows users to consider essentially any candidate ingredient, as long as data on nutrient content, technical parameters, and prices are available. A preliminary step in the RUSF development process was conducting a price survey of all potential local ingredients that could be used in a formulation. An initial list of 89 possible locally-available candidate ingredients was identified through the following multi-step, systematic approach:

1. National statistics on production and importation of cereals/grains, legumes, nuts, seeds, milk products, sweeteners, and oils were compiled from the Food and Agriculture Organization (FAO) and African Development Bank (ADB). All ingredients that had reported production and/or import volumes above the defined locally-available cutoff were added to the list.
2. It was assumed that the agricultural commodity markets would be similar between Ghana and Sierra Leone since both countries are located in West Africa; therefore, locally-available candidate ingredients from a previous model used in Ghana not represented in FAO and ADB statistics were added to the list.
3. Any additional candidate ingredient requests from the food technologists not previously included were added to the list.
4. A few additional commodities were identified as possible candidate ingredients based on a wholesale market assessment after visiting two large markets in Freetown.

After this draft list was developed, additional ingredients were excluded based on feedback from local producers, nutritionists, food technologists, Sierra Leone government ministries, and other local experts. A subsequent list of 27 locally available candidate ingredients remained following these additional exclusions. The final candidate ingredient list for the Sierra Leone model consisted of 53 ingredients (Table 1).

**Table 1:** Candidate ingredient list of locally produced and imported foods

Ingredient	Source Designation
Cereal/Grain, Bulgur	Locally-Available
Cereal/Grain, Cous	Locally-Available
Cereal/Grain, maize, dried, raw	Locally-Available
Cereal/Grain, Maize, yellow, flour of whole-grain	Locally-Available
Cereal/Grain, millet, Pearl, whole grain, raw	Locally-Available
Cereal/Grain, rice flour, white	Locally-Available
Cereal/Grain, rice, brown	Locally-Available
Cereal/Grain, Rice, Local Mende	Locally-Available
Cereal/Grain, Rice, White	Locally-Available
Cereal/Grain, Sorghum	Locally-Available
Cereal/Grain, wheat flour, whole grain	Locally-Available
Copalis Nutripeptin	Imported
Imperial-Oel-Import Handelsgesellschaft mbH Maris Powder ED 0106	Imported
InnoBio Algae Oil Powder	Imported
InnoBio InnoMega Alpha Linolenic Acid 80% EE	Imported
Kelatron Corporation CALCIUM RICE CHELATE 30%	Imported
Legume, Beans, Broad beans (Vicia faba)	Locally-Available
Legume, beans, chickpea flour	Imported
Legume, Beans, Cowpea, (Vigna spp.)	Locally-Available
Legume, Beans, Dry, Other, […] (Phaseolus vulgaris)	Locally-Available
Legume, Beans, Pigeon pea, […] (Cajanus cajan; C. indicus)	Locally-Available
Legume, Groundnut flour, defatted	Imported
Legume, Groundnut paste	Imported
Legume, groundnut, Bambara groundnut, dried, raw	Locally-Available
Legume, groundnut, high oleic, roasted	Imported
Legume, groundnut, shelled, local PPB source	Locally-Available
Legume, Soy flour, defatted	Imported
Legume, Soy protein concentrate, crude protein basis (N × 6.25), produced by acid wash	Imported
Legume, Soy protein isolate	Imported
Microalgae/whole cell algae/chlorella/blue green algae	Imported
Milk, acid casein	Imported
Milk, acid whey powder	Imported
Milk, caseinate, calcium	Imported
Milk, dry, nonfat, regular, without added vitamin A and vitamin D	Imported
Milk, dry, whole, without added vitamin D	Imported
Milk, Fatfilled, dry	Imported
Milk, Milk protein concentrate	Imported
Milk, Reduced lactose whey	Imported
Milk, rennet casein	Imported
Milk, sweet whey powder	Imported
Milk, whey protein concentrate 34	Imported
Milk, whey protein concentrate 80	Imported
Milk, whey protein isolate	Imported
NuMega Fish Oil Powder	Imported
Oil, Canola, Rapeseed	Imported
Oil, Fish oil, menhaden	Imported
Oil, Groundnut	Locally-Available
Oil, Linseed	Imported
Oil, Maize	Imported
Oil, Palm	Locally-Available
Oil, Palm kernel	Locally-Available
Oil, Soybean	Locally-Available
Oil, soybean, high oleic, Plenish	Imported
Seed, Melon	Locally-Available
Seed, sesame flour, partially defatted	Imported
Seed, Sesame seed (Sesamum indicum)	Locally-Available
Spirulina	Imported
Sugar, brown	Locally-Available
Sugar, white	Locally-Available
Tuber/Root, Cassava flour	Locally-Available
Tuber/Root, Cassava, gari	Locally-Available

Using the LP tool designed for Sierra Leone, preliminary formulations were generated in the Food and Nutrition Food Lab at Washington University School of Medicine (WUSTL). Four potential RUSF formulas were created at WUSTL that contained peanuts, milk powder, vegetable oil, sugar, and a vitamin mineral complex. The major difference between the four was the indigenous ingredient present: millet, split pea, cowpea, bambara bean. These four potential RUSF formulas were then tested informally by pregnant women at an antenatal clinic in Pujehun, Sierra Leone. Informal acceptability showed the formula with millet was the overwhelming favorite. The millet formula was further optimized for nutrient content, feasibility of production and organoleptic acceptability. Rudimentary blending and heating techniques were used to create a homogenous lipid matrix containing a variety of legumes, cereals, milk powders, vegetable oils, sugar, vitamin mineral complex and an emulsifying agent until the final formula was determined.

Table 2 specifies the ingredient composition of the study RUSF, named *Mama Dutasi*. The RUSF will provide a total of 520 kcal, 18 g protein, 200% of RDA for most micronutrients during pregnancy (Table 3). The supplement has also been optimized to provide excellent protein quality. The formulation contains an optimal polyunsaturated fatty acid composition by reducing the percent of energy from linoleic acid (omega-6) and increasing the percent of energy from α-linolenic acid (omega-3) to 4.5% and 1.5%, respectively [36–38].

**Table 2:** RUSF Ingredient Composition

Ingredient	Amount (g)
Cereal/Grain: Whole, raw pearl millet	7.500062
Milk: Dry, nonfat without added vitamin A and vitamin D	21.49301
Milk: Whey protein isolate	6.832923
Palm oil	2.160523
High oleic soybean oil	25.64348
Brown sugar	20
Legume: Shelled groundnut	10

**Table 3:** Nutrient profile of study foods

Nutrient (units)	RUSF	CSB+ with sugar, oil. Iron/folic acid^b^	RDA for pregnancy, 19–30 yr	UL (Tolerable Upper Limit Intake)
Energy, Kcal	520	1177		
Protein, g	18	35		
N-6 (% from energy)	4.5	7.3^c^		
N-3 (% from energy)	1.5	.7^c^		
Vit A (mcg)	770	2621	770	3000
Vit B1/Thiamine (mg)	2.8	0.5	1.4	
Vit B2/Riboflavin (mg)	2.8	3.5	1.4	
Vit B3/Niacin (mg)	32	20	18	35
Vit B6 (mg)	3.8	2.5	1.9	100
Vit B12 (mcg)	5.2	5.0	2.6	
Folic acid (mcg)^a^	500	275	400	1000
Vit C (mg)	170	225	85	2000
Vit D (IU)	1200	1104	600	4000
Vit E (mg)	30	20.8	15	1000
Iron (mg)	30	66.5	27	45
Zinc (mg)	22	12.5	11	40
Calcium (mg)	1600	905	1000	2500
Chromium (mcg)	60		30	
Copper (mcg)	2000		1000	10,000
Iodine (mcg)	300	100	220	1100
Magnesium (mg)	300		350	350
Selenium (mcg)	120		60	400

^a^ Amount recommended for supplementation, with 200 mcg to come from dietary sources for total RDA of 600 mg/d

^b^ Daily ration: 250 g supercereal (CSB+) with sugar, 20 ml oil, 60 mg Fe supplement with 400 mcg folic acid

^c^ Calculated based on a WFP supercereal product containing ~ 64% corn, ~ 24% soybean,~ 20 ml vegetable oil

Large scale production of this product requires the millet be in pre-processed form. The most effective pre-processing methods are roasting or extrusion cooking followed by milling. In this instance, the process used was sorting, drying, roasting, and milling. Pre-processing was performed at FINIC Industries in Kissy, Sierra Leone. The following outlines the pre-processing of millet in more detail:

##### Sorting

Proper sorting and cleaning is a necessary step to all raw commodities regardless of the harvest process. Sorting/cleaning the millet required removing dirt, debris, and stones. Visible debris like twigs and grass were removed by hand while dirt and stones were removed with a de-stoner. Once cleaned, the millet was allowed to drain.

##### Roasting and drying

Roasting is a dry heat method which bakes/toasts the ingredients. The whole grains will be placed in a rotary oven heated by charcoal and dried for 12+ hours. This process is used to destroy microorganisms, lower the water activity, inactivate enzymes and catalyzes flavors via browning reactions.

##### Milling

Milling or size reduction of whole grains was necessary prior at incorporating into the process line as to not damage the existing equipment. Size reduction of ingredients is also important for edibility. Although grinding of the final product occurs during the production process for particle reduction and homogenization, it is important that the millet have a small enough particle size to not damage the grinder plates.

The roasted, cooled, dried millet was ground to a particle size similar to baking flour and packaged in clean lined peanut buckets. The millet flour was cleared by quality control and food safety testing for microbial contamination at Eurofins Scientific Inc., Des Moines, Iowa, USA and was stored until production of the RUSF product.

The final product was packaged in 100 g foil sachets and identified with a custom *Mama Dutasi* label. Analytical testing was performed for food safety and quality assurance for aflatoxin and microbial contamination at Eurofins Scientific Inc., Des Moines, Iowa, USA.

#### Control group food

The control group will receive the standard of care for malnourished pregnant women in Sierra Leone which is 3.5 kg super cereal with 350 mL vegetable oil every two weeks. This provides 250 mg portion per day of the super cereal and 25 g oil per day for the mother. Iron and folic acid supplement goal is 90 pills through pregnancy.

The study will assure that women have an uninterrupted access to the ready to use local supplementary food and super cereal if they are in the control group. Table 3 provides a comparison of the nutritional composition of the study RUSF, standard of care food, and recommended daily intake for pregnancy. Women will receive the food for the duration of their pregnancy.

### Interventions to reduce inflammation

The intervention group will receive a package of five interventions aimed at decreasing the burden of infections in pregnant women in order to decrease the risk of stillbirth, fetal growth restriction, and premature delivery. This package will include an insecticide-treated mosquito net at the time of enrollment, sufadoxine-pyrimethamine given every 4 weeks beginning at 13 weeks gestation, azithromycin given at the time of enrollment and at 28–34 weeks of gestation, single dose of albendazole at enrollment, and testing and treatment for bacterial vaginosis at enrollment and again in the third trimester. Table 4 details the anti-inflammatory interventions.

**Table 4:** Anti-Inflammatory Interventions, Dosing, and Timing of Administration

Intervention	Dose	Time of administration
Insecticide-treated mosquito net	n/a	• Enrollment
Sulfadoxine-pyrimethamine	1500 mg/ 75 mg	• Enrollment or 13 weeks gestation, whichever is later • Every 4 weeks until delivery
Azithromycin	1 g	• Enrollment or second trimester, whichever is later • Weeks 28–34
Albendazole	400 mg once	• Enrollment or second trimester, whichever is later
Testing and treatment for bacterial vaginosis with metronidazole	750 mg PO daily for 7 days	• Enrollment • Weeks 28–34

The control group will receive the current recommendations of the government of Sierra Leone, which includes 60 mg iron and 400 μg folic acid supplementation starting at enrollment, an insecticide-treated mosquito net at the time of enrollment, three doses of sulfadoxine-pyrimethamine during second and third trimester, and a single dose of albendazole for deworming at enrollment.

Table 5 provides a summary of the schedule of activities and interventions for subjects by week of study participation.

**Table 5:** Schedule of activities/ interventions for subjects by week of study participation

	Study Period									
	Enrollment									
Week	0	2	4	6	8	Third trimester	Delivery	6 weeks post-partum	3 months post-partum	6 months post-partum
Enrollment:										
Eligibility Screen	X									
Informed consent	X									
Socio-demographic questions	X									
Assessments:	X				X					
HFIAS^a^, Food frequency questionnaire	X				X					
Clinic visit for intervention and control food supply	X	X	X	X	X					
Adherence/educational messages	X	X	X	X	X					
Surveillance of clinical events	X	X	X	X	X					
Maternal anthropometric measurements	X	X	X	X	X					
Infant anthropometric measurements							X	X	X	X
Interventions:										
Food distribution	X	X	X	X	X	X				
Bed net distribution	X									
Testing and treatment of bacterial vaginosis	X					X				
Albendazole (400 mg)	X									
Azithromycin (1 g)	X					X				
Sulfadoxine/ pyrimethamine (1500 mg/75 mg)	X		X		X	X				

^a^*HFIAS* household food insecurity assessment scale

Food Safety specifications: RUSF specifications will be in accordance with the CODEX guidelines for supplementary food products [39].

### Data management and analysis

Clinical data including demographics, anthropometry, morbidity, and mortality data will be collected by field workers using standardized forms. Field workers will be trained in the questionnaires and measurements prior to collecting any data. Completed data forms will be stored in a secure locked central location. All data will be double entered, compared, and sealed in a password-protected electronic database before the randomization code is broken. All data discrepancies will be resolved by examination of the original data cards and discussion with the relevant field workers. The data set will be locked after all discrepancies have been resolved.

### Statistical analysis

Descriptive statistics will be used to characterize the population. Student’s t-test will be used to assess whether primary outcomes of infant birth weight and length, and secondary outcome of maternal weight gain improved significantly in the intervention group compared with the control. Fisher’s exact test will be used to determine if the proportion of mothers recovering from malnutrition and of infants with LBW are improved in the intervention group. Regression modeling will be used to investigate factors that influence maternal and infant outcomes. Independent variables to be considered as covariates for inclusion in the models include: maternal age, weight, number of previous pregnancies, anemia, illness and number of weeks of intervention. For longitudinal measures, the groups will be compared using repeated measures mixed model analysis of variance. Covariates will include age, number of previous pregnancies, years of education and clinic location.

### Dissemination

Findings of this study will be disseminated through publication in peer reviewed journals and presentations at national and international conferences. The findings will also be shared with the Director of Nutrition for the Ministry of Health and Sanitation for the government of Sierra Leone. Study results will be presented to the communities and participants involved through community meetings and information sessions.

## Discussion

Maternal malnutrition remains a major problem in low- and middle-income countries, resulting in high maternal and neonatal morbidity and mortality. Prevention and treatment of maternal malnutrition is vital to reduce stunting, improve cognitive outcomes, and maximize professional achievement [3–5]. The risk of stunting and wasting has been shown to be increased in small-for-gestational age infants, suggesting that pregnancy interventions are vital [40]. Prenatal factors appear to be more important than postnatal factors for preventing stunting [41, 42]. Prior studies of nutritional intervention during pregnancy have demonstrated reduction in preterm birth and increased birth weight but have not improved stunting in the postnatal period [43]. If indeed this intervention results in longer and larger infants at birth in Sierra Leone, this will be the first time a combined intervention of RUSF and bundled anti-infective therapies has resulted in improved birth outcomes. This could serve as a model for future national programs, as well as, guide international non-governmental organizations.

One limitation of the study is the lack of blinding of the nutritional and anti-infective interventions between the two groups. The study RUSF and standard of care flour are distinctly different in packaging and appearance. Those participants randomized to the intervention group will receive more obvious anti-infective interventions during antenatal visits. Mitigating the un-blinded nature of the intervention distribution is that choice of primary outcome, birth length, will be obtained in a standardized, objective manner. In order to account for this lack of blinding, study managers will be blinded to treatment during data analysis. Another limitation is the possibility that participants will share or trade their assigned food. Participants will be educated about the need to comply with the assigned intervention. We anticipate this will be an unlikely occurrence as past research by our group of compliance with supplementary foods in Malawi rarely demonstrated sharing or trading of assigned foods [44]. An additional limitation of our data will be that the control group will be receiving the standard of care for Sierra Leone, but these interventions are not universally adopted, especially in very rural areas such as Pujehun. This may result in improved outcomes in the control group than would be expected from currently available data for pregnant women and infants.

### Trial status

Recruitment of trial participants began in March 2017. Follow-up with study subjects is expected to last 24 months. The study is currently enrolling participants. There have been no prior publications involving this study or study data.

## Abbreviations

HFIAS: Household food insecurity access scale
MUAC: Mid-upper arm circumference
RUSF: Ready to use supplementary food
